# Protocol for a pragmatic randomised controlled trial to evaluate effects of a brief intervention for emergency department attendees who present with moderate or high levels of non-specific psychological distress: a pilot study

**DOI:** 10.1186/s40814-015-0028-9

**Published:** 2015-09-29

**Authors:** Petra Lawrence, Paul Fulbrook

**Affiliations:** 1 Nursing Research and Practice Development Centre, The Prince Charles Hospital and Australian Catholic University, Brisbane, Australia; 2 School of Nursing, Midwifery and Paramedicine, Australian Catholic University, Brisbane, Australia

**Keywords:** Psychological distress, Emergency department, Screening, Motivational interview, Telephone

## Abstract

**Background:**

Screening and brief intervention in the emergency department (ED) has almost exclusively focused on individuals with alcohol-use problems. The early detection of mental health problems before problems become severe will enable early intervention and support which may improve health and prevent further deterioration. The main aim of this pilot study is to provide evidence of the acceptance of a telephone intervention aimed at ED attendees with moderate or high psychological distress. This will be determined by recruitment rates, retention rates and participant satisfaction with the intervention. Secondary outcomes include whether socio-demographic variables have an impact on retention rates, and whether the intervention had any impact on psychological distress.

**Methods/Design:**

This study will be a single-site pragmatic randomised controlled pilot study. Consenting ED attendees will be screened with the Kessler Psychological Distress Scales (K10). There will be three arms to the study: a moderate/high psychological distress group with or without intervention, and a low psychological distress group. Those with severe psychological distress will be excluded. All included participants will be followed-up at 1, 3, 6 and 12 months post-recruitment. Retention rates will be determined by successful completion of surveys at the follow-up time-points. Psychological distress will be measured by the K10 at all follow-up time-points.

**Discussion:**

This study will provide information regarding the potential for screening and recruitment at an opportunistic hospital presentation. It will provide data for a future larger study with regard to participants accepting to be included in this study. Participant acceptability will be measured in terms of recruitment rates and retention rates measured by successful follow-ups over the following 12 months post-recruitment.

**Trial registration:**

Australian and New Zealand Clinical Trials Registry ACTRN12614000031662. Registered 10/01/2014.

## Background

Mental illness is a major public health issue due to morbidity and other associated costs [1]. However, there are also individuals who may be free from a diagnosable mental illness but who may not feel healthy and/or are functionally impaired [2]. These sub-threshold symptoms are significant due to their prevalence, clinical significance, costs and risk of progression to more severe symptoms [3]. The ED is a potentially effective setting to target these issues due to the high prevalence of mental health problems in attendees [4–9]. Detection of mental health problems and treatment seeking before problems become severe, may improve health and prevent further deterioration [10]. This is consistent with the recommendations from several Australian government reports and publications regarding mental health and its management [11, 12].

The World Health Organization (WHO) describes mental health as being more than an absence of a mental disorder. It is a state of well-being, where the individual flourishes and realises their own potential, can deal with normal life stressors, work productively and contribute to society where they live [13]. Mental health can therefore be described as a condition free from mental illness, whilst mental illness describes symptoms of insufficient mental health. In Australia, almost half of the adult population (7.3 million) will experience a mental illness at some point in their life, whilst 20 % (3.2 million) will experience a mental illness this year, the most common being depression and anxiety [14]. However, there are many people in the community who, despite being free from a diagnosable mental disorder, may be languishing and not leading productive and healthy lives [2]. In 2003, mental health disorders contributed to 13 % of the total disease burden in Australia [15], and its annual cost is approximately $20 billion due to loss of productivity and reduced workforce participation [16]. In a 12-month period, almost 12 % of the Australian adult population made use of services for mental health problems, and from this group, only 35 % met the criteria for a mental health disorder, and a small proportion (6.1 %) of people with no mental health disorder also made use of these mental health services [17]. This reveals that there are other indicators of need for mental health services rather than mental illness alone.

Although sub-threshold syndromes are less defined than diagnosable mental illness, they still pose serious problems from psychological distress, which can impair a person’s development, career and education opportunities and increases their risk of future mental illness [18, 19]. The Australian Bureau of Statistics (ABS) uses the K10 to determine levels of non-specific psychological stress in its population surveys. Studies involving the K10 reveal a strong association between very high levels of psychological distress and diagnosable mental illnesses such as anxiety and depression [20]. The ABS National Survey of Mental Health and Wellbeing 2007 revealed that 67 % of the population had low-level psychological distress, 21 % had a moderate level, 9 % had high level and 4 % had a very high level [14, 21]. The absence of mental illness does not necessarily mean the presence of flourishing mental health, and it is important to consider the risk of sub-threshold symptoms progressing from moderate symptoms to more severe disorders [3, 18]. The promotion and protection of mental health may be beneficial and more cost effective, rather than to alleviate mental illness [10].

Early interventions and the recognition of the spectrum of mental health issues including those with mild or moderate impact with high and low prevalence were identified as a key area for reform in Australia's 4th National Mental Health Strategy [11]. The National Report Card on Mental Health and Suicide Prevention [12] also states that increased access to timely and appropriate health services reduces the longer term need for crisis intervention. Consumers can access mental health services either through hospitalisation, residential care, outpatient services or community services. From the 12 % of the population who accessed mental health services, one-third consulted community-based providers, mainly general practitioners (GP) who generally managed problems such as anxiety, depression and sleep disturbances [22]. However, GPs may have limited time in their practice as well as limited training and experience with mental health disorders [23]. EDs also provide some mental health services, but usually for patients who have an urgent or semi-urgent need [24], and the number of people accessing mental health services through the ED is increasing. In 2011–12, from the total of 7.8 million [25] presentations to public EDs, there were 248,501 [24] mental health-related presentations, representing 3.2 % of all presentations. This is an increase from 2008–2009, when there were 172,000 [26] mental health presentations from a total of over 7.2 million [27] ED presentations, representing about 2.4 % of all ED presentations.

Some EDs in major hospitals have developed an increasingly important role in providing crisis services to patients with mental health issues [28], and it is considered an appropriate setting for the detection of mental health problems [29]. Considering that the ED has a high yield of attendees with mental health problems, and that in the community, 30 % of the adult population are currently experiencing moderate and high levels of psychological distress, the ED would seem an appropriate setting for the detection of mental health problems in a non-mental health treatment-seeking population. Screening and identification of ED attendees with moderate or high psychological distress and encouraging them to seek follow-up care and support may improve health outcomes, and further deterioration of symptoms may be prevented.

### Prevalence study

A single-site cross-sectional study (*n* = 708) was conducted in 2011 to establish the prevalence of mental health issues of ED attendees [30]. Several mental health measures were employed, including the K10. Our data revealed that only 18 participants (2.6 %) received a primary ICD diagnosis related to mental health whereas 10.1 % scored in the very high K10 distress category. We also included a question on our general demographic and general health survey asking whether patients had any “mental health issues?”, and almost 17 % of participants answered positively. Based on norm data from the Australian population and the observed K10 scores from our pilot study, we were able to calculate the probability of ED attendees having a mental health disorder [31]. Stratum-specific likelihood ratios were applied to the sample of the 708 attendees. It was found that 37 % of all participants may have had an actual mental health disorder, which is higher than the population norm. Our data also showed that almost 40 % of ED attendees were affected by moderate/high non-specific psychological stress (identified using K10) [30]. This latter group is our target sample for the proposed study (moderate/high psychological distress).

### Current evidence

#### Motivational interviewing

Motivational interviewing (MI) is a non-confrontational, client-centred, directed therapy, which prepares individuals to become more receptive to change by exploring dissonance in the perceived benefits and costs of behaviours [32–34]. MI was developed originally for the treatment of substance-use [32], and its central principle is that motivation to change should be elicited from people, not somehow imposed on them [35]. It is an approach used to help a client realise that they may have a problem, build commitment to treatment, increase their engagement in treatment and enables behaviour change. The stages of change model [36] has proved useful for the understanding and conduct of an MI session. All change is preceded by some degree of ambivalence [35]; however, MI is particularly useful for working with clients who are ambivalent, resistant or reluctant to change [32, 37].

In the ED, MI has been shown to be effective in reducing alcohol consumption [38–43]. There are several studies which use MI as a pre-treatment to encourage treatment-seeking behaviours and therapy engagement. These studies have been set in an inpatient environment [44, 45], medical centres for veterans [46, 47], specialist outpatient clinics [48, 49] and a university psychology clinic [50]. All studies delivered the MI face-to-face, except for Seal et al. (2012) and Zinjani et al. (2008) which delivered the MI by telephone. The studies have demonstrated that participants randomised to MI pre-treatment had an increased attendance to psychiatric appointments. MI as a pre-treatment has also been shown to help reduce symptoms of worry [51] and fear [48]. The inclusion of MI to treatment strategies has benefits; however, efficacy has only been demonstrated on patients with diagnosed mental illness. Only one other study has been found which focused on participants with mild and moderate distress [10]. This study did not use MI but acceptance and commitment therapy and mindfulness to promote positive mental health. The authors stated that they used clinical judgement to identify patients and did not document whether they also used a screening tool. So far, we have found no studies which focus exclusively on MI with a sample with lower severity mental health problems.

The proposed pilot study focuses primarily on participants with moderate or high levels of psychological distress (identified by the K10) due to the high prevalence and excludes those with very high levels of psychological distress as this may indicate a pre-existing mental health disorder [20]. The purpose of the MI is to promote early intervention and to motivate participants to seek assistance for psychological distress. This study will also trial the provision of telephone MI which has been shown to be effective in samples with severe mental illness [46, 47] and alcohol studies based in the ED [42, 43]. In summary, there is extensive evidence of the effectiveness of brief intervention (MI) in the ED for alcohol-related samples, but it is relatively untested in mental health populations.

#### Aim

The main aim of this pilot study is to provide information for the planning of a future larger trial. The socio-demographic characteristics of participants and their relationship with recruitment rates and attrition rates will be assessed. Satisfaction with the intervention will also be assessed.

Secondary aims are to assess whether the telephone intervention has an effect on psychological distress levels and to determine if the K10 is a suitable method of screening and monitoring psychological distress. This is an unfunded PhD study which will inform the viability of applying for a substantive grant for a larger randomised controlled trial (RCT) with an economic evaluation.

## Methods/Design

Pragmatic randomised controlled pilot study.

### Ethical approval

Ethical approval for this study has been provided by the Human Research Ethics Committee from Metro North Hospital and Health Service (ref: HREC/13/QPCH/244) and the Australian Catholic University Human Research Ethics Committee (ref: 2013 294Q).

### Participants

All adult patients presenting to the ED of a public hospital during the specified data collection period will be screened to participate in the study. Those who meet the screening criteria and consent to participate in the study will be able to enter the pilot RCT.

Based on psychological distress assessment, consenting ED patients will be categorised into three initial groups; those with:

1. Moderate or high psychological distress
2. Low psychological distress
3. Very high psychological distress

Subsequently, group 1 will be randomly allocated to receive either the MI or usual care (usual care does not involve MI). A randomised sample of participants which report low psychological distress will form group 2 to represent a “low-stress” population control group. The inclusion of a “low-stress” control group will allow for further assessment of recruitment strategies and retention of participants. Group 3 will be excluded from the study (but will be provided with advice to contact a health professional). Thus, there will be three arms to the study (see Table 1).

**Table 1:** Randomisation of groups

Condition 1		Intervention
Group 1	Moderate/high psychological distress	MI plus usual care
	Moderate/high psychological distress	Usual care
Group 2	Low psychological distress	Usual care

#### Inclusion criteria

Inclusion criteria will include all alert and orientated English-speaking adults (over 18 years of age) who present to ED. Those attendees who have moderate or high levels of non-specific psychological distress, identified by the K10 (score 16–29) and do not require hospital admission will be eligible to enter the RCT study arm. They will be randomly allocated to either the intervention or control groups. Of the remaining participants, those with low psychological distress (score 10–15) will be enrolled in the “low-stress” control group.

#### Exclusion criteria

Participants with very high K10 scores will be excluded from the study and will be given phone numbers of community mental health clinics. Other exclusions include ED attendees unable or unwilling to give consent or those unwilling to be contacted by telephone, people with a cognitive impairment or a learning disability, participants admitted to hospital as in-patients or already participating in mental health programmes. Participants will also be excluded if they are in police custody.

### Screening

All participants will be screened using the K10. The K10 has been used in WHO surveys, with over 200,000 participants across 26 countries, as well as US, Canadian and Australian surveys [52]. It is a self-report tool that was developed based on extensive psychometric analysis in a large general population sample and was derived from existing screening scales by applying item-response theory to identify items that produced maximal discrimination of respondents at the 90–99th percentile, with a focus on severe mental illness [53, 54]. The resulting scale produced high discrimination scores between community and non-community cases of Diagnostic and Statistical Manual of Mental Disorders (DSM)-defined psychiatric disorders and had excellent discrimination in severe cases. The purpose of the screening scales is to screen for broadly defined mental disorders rather than for one particular diagnosis [53].

The K10 requires respondents to identify the frequency of symptoms of psychological distress within the past 30 days and focuses on anxiety and depressive states. It comprises four questions regarding anxiety, which focus on agitation and nervousness, and six questions about fatigue and negative effects. Each item is scored using a 5-point scale, ranging from 1 (none of the time) to 5 (all of the time), that defines behavioural, emotional, cognitive and psychological manifestations [55]. Participants categorised with low distress (score 10–15) are likely to be well. Those with moderate distress (score 16–21) are likely to have a mild mental health disorder, whilst those with high distress (score 22–29) are likely to have a moderate mental health disorder. Those scoring very highly (score 30–50) are likely to have a severe mental disorder [21]. There is a strong association between very high K10 scores and a current Composite International Diagnostic Interview (CIDI) diagnosis of anxiety and affective disorders and a lesser but still significant association between other mental health categories or the presence of any current mental disorder [20].

It could be argued that people coming into the ED would have higher distress scores due to the nature of their presentation. However, the K10 is assessing psychological distress over a 30-day period and not distress experienced on the day of presentation to ED or the few days immediately prior.

### Intervention

In this study, the MI will be used to encourage and motivate study participants to seek and obtain further assistance for their psychological needs. The overall spirit of MI is described as collaborative and evocative, and honours patient autonomy. A MI follows four guiding principles: resisting the fighting reflex, understanding and exploring the patient's own motivations, listening with empathy and empowering the patient, and encouraging hope and optimism [56]. The MI intervention has been designed to be pragmatic in that it will be tailored to each participant’s individual circumstances and needs.

Following recruitment, all participants will be provided with standard care, i.e. usual care from their ED attendance. Participants who are randomised into the intervention arm will receive an initial MI, delivered by telephone interview 48–96 h after their ED attendance, with up to three additional MIs by telephone during the following 2 weeks. Each MI is expected to last up to 60 min (a total of not more than 4 h for each study participant).

### Follow-up

For all study participants, longitudinal follow-up will occur at 1, 3, 6 and 12 months by telephone interview. The purpose of a 12-month follow-up is to ensure the usefulness of longitudinal data by measuring the impact of the intervention over time [57]. We also want to track the natural course of mental health from all participants. The primary goal of using the K10 at each follow-up time-point is to measure changes in psychological distress over 12 months and to be able to compare data with other studies.

### Outcomes

The main outcomes

- recruitment rates as a percentage of eligible participants
- attrition rates by measuring the completion of follow-up data
- participant satisfaction by measuring whether the intervention is acceptable to the participants

Secondary outcomes

- measurement of the demographic characteristics of participants recruited to each arm such as: age, gender and education to determine inequities in retention rates
- determining whether the intervention had an effect on K10 scores

### Sample size

There is a limited amount of published data regarding the ideal size for pilot studies, and it has been commented that it seems that sample calculations may not be required for this type of study [58, 59]. An audit of registered studies found that the median sample size per arm for pilot studies was 30 (range from 8 to 114) [59]. Based on this evidence, we will recruit the median sample of 30 participants per arm.

### Recruitment and randomisation

#### Recruitment

All adult attendees who present to the participating ED and meet the inclusion criteria will be eligible to enter the study. Recruitment of study participants will occur in the ED by a research assistant (RA), which will ensure that the existing staffing levels at the ED research site are not affected. Recruitment will occur at the time of the patient’s presentation to ED, with due consideration given to their particular circumstances. Those who are indisposed, severely injured or severely distressed due to their injuries may not be approached; in such circumstances, guidance will be sought from attending ED staff on an individual basis.

Each potential participant will be provided with an information letter by a researcher, explaining the study. Those who agree to participate will be required to provide written consent.

#### Randomisation

Attendees who have moderate or high psychological distress will be randomised into intervention and control arms. Those with low psychological distress will be randomly selected (simple randomisation) to form a low distress comparison group for the purpose of measuring retention rates at follow-up and changes in K10 scores over time.

#### Allocation concealment

Randomisation to groups will be done with computer-generated number tables. A stratified randomisation method will be used. Screened participants will be allocated to groups using a balanced block design to ensure that there are equal numbers of participants in the intervention and control group. A separate randomisation list will be drawn up for each of the health professionals (strata) that will be delivering the MI, to ensure that there are equal numbers of participants managed by each interviewer. This method will be used rather than a remote service to simplify procedures in the busy ED setting. The participant will not know at baseline whether they will have the MI intervention. Potential MI participants will be allocated by the health professional to treatment group or non-treatment group using an experiment to control a ratio of 1:1.

Follow-up at the designated time-points will be conducted by a RA who is not involved with data collection and will be blinded to the participant’s group allocation.

### Data collection

Data collection will be completed in randomly selected 5-h blocks (5 per week/1 per day) on randomly selected days between the hours of 0700–2200 (0700–1200, 1200–1700, 1700–2200) until the required sample size has been achieved. These periods have been selected based on the presentation patterns found in our prevalence study.

Participants will initially be contacted by a RA when they are admitted to the ED, and this contact will primarily be concerned with consent screening and baseline data collection. Participants will be approached by the RA whilst they are in the ED. The RA will not be present when the participants complete the assessments. Paper surveys will be in the control of the RA at all times whilst in the ED and kept in a locked filing system. ED staff will be blinded to the results.

Data will be initially logged into an Excel database then transferred to SPSS version 21, on a computer which can only be accessed by password which will be known only to investigators. Paper surveys will be kept in a secure filing cabinet in a lockable office for a minimum period of 5 years from the date of publication as per National Health and Medical Research Council guidelines [60]. Data entry will be checked for inconsistencies and errors by the research team.

### Intervention

The MI will be conducted by the telephone. The MI health professionals (one senior psychologist and three advanced-practice mental health nurses) will be provided with training sessions through an accredited training provider regarding the “spirit” of motivational interviewing. Clinical performance will be monitored by evaluating a random sample of interviews. MI will be assessed using the Motivational Interviewing Treatment Integrity 3.1.1 (MITI 3.1.1) [61]. Feedback of performance will be provided as necessary.

### Measures

The main outcome of this pilot trial is the identification of recruitment and retention rates from baseline to all follow-up time-points. Table 2 summarises the measures, instruments and their administration timetable. Instruments have been carefully selected due to their specific relevance to the population of interest. General measures have been selected due to their widespread use, robust reliability and validity testing and availability of norm values [54, 62–67].

**Table 2:** Measures

Measure	Instruments	T1a	T1b	T2	T3	T4	T5
		Screening	Baseline				
Demographics	Demographics	X	✓	X	X	X	X
Mental health and well-being	K10 [54]	✓	X	✓	✓	✓	✓
	DASS 21 [62].	X	✓	X	X	✓	✓
	Satisfaction with Life Scale [63].	X	✓	✓	X	X	✓
	WHO-BREF [64].	X	✓	X	✓	X	✓
Moderators	Readiness to change stage [65].	X	✓	X	X	X	X
	General self-efficacy scale [66]	X	✓	✓	X	X	✓
Outcomes	Health service usage and health-related actions survey [67].	X	✓	✓	✓	✓	✓
	MI satisfaction questionnaire	X	X	✓	X	X	X

#### Baseline

At recruitment, all consenting participants will be screened at time-point-a (T1a) and either included or excluded depending on K10 scores. Time-point-b (T1b) will commence post-screening, and data will then be collected from all eligible participants. Other data (such as primary ED diagnosis) will be collected from patient’s medical records.

#### Follow-up

Satisfaction of MI will be assessed with a specifically designed tool 5-item Likert scale questionnaire.

Pre- and post-intervention measures will be used to assess outcomes including motivation, confidence and health-seeking behaviour and utilisation, mental health and well-being and subjective quality of life.

All participants will be followed-up at four time-points: 1 month (T2), 3 months (T3), 6 months (T4) and 12 months (T5). Data will be collected by a registered nurse RA via telephone. Data will be input initially via onscreen software, into an Excel database, where they will be checked and verified for transcript errors before importing into an SPSS (version 21) database for analysis.

### Data analysis

Attrition rates will be measured by successful follow-up and completion of a telephone survey at each time-point. Due to the small sample size of the pilot study, and therefore being underpowered to detect change between groups [68], descriptive data will be used to analyse patient characteristics and socio-demographic data, using measures of central tendency to measure sample distribution and spread of the data (mean and standard deviation, median and percentiles), and confidence intervals. Percentages will be used for categorical variables. We will also compare to those who are lost to follow-up. Data analysis will be performed using SPSS (v 21). We will use regression/ANCOVA analysis to compare variables such as age, gender, and education on secondary outcomes at the different follow-up time-points. However, the results from this analysis must be viewed with uncertainty due to the small sample size. Participants lost to follow-up will be excluded from later analysis at follow-up time-points and basic socio-demographic data and K10 scores used to describe this group.

## Discussion

There is evidence regarding the effectiveness of screening and intervention in the ED for people with alcohol-use disorders [38–43]. However, screening and intervention in the ED for mental health problems has not been well investigated and has the potential to improve the quality of life of people who have moderate and high levels of psychological distress who might not otherwise seek mental health services. This project will provide evidence to inform the design of a larger study in the form of measurable outcomes regarding recruitment rates, retention rates at follow-up and satisfaction with the telephone intervention. The findings will enable practical questions to be answered about the most effective way to implement and recruit for a future study which will test the intervention on a larger scale. The findings will also provide a prospective longitudinal study of the natural history of at-risk and vulnerable population groups after their ED attendance and also information about the mental health of those who reported low levels of psychological distress at baseline.

Systematic screening of ED patients has the potential to bring about a major change in environment and culture. Currently, there is no routine follow-up of these attendees. Thus, it is possible that many of them—if they are not supported—will experience deterioration in their mental health, which will place an increased demand on future health services. Furthermore, for some patients, their mental health deterioration may lead to crisis situations such as extreme anxiety and depression and the possibility of other behaviours. Therefore, the longitudinal follow-up is essential to measure these possible changes.

Our project is unique in that it will pilot test an intervention that is not well tested in mental health contexts, and which is both practical and sustainable, and has the potential to integrate services from several healthcare disciplines. The project is targeted at the delivery level of healthcare services, focusing primarily on preventative health care and mental health promotion. Whereas previous research has focused on a single lifestyle problem group, e.g. alcohol users, or those with severe mental health problems, this study will focus on those with moderate and high psychological distress. In this context, we have found limited research to date that has investigated this population.

## Trial status

The trial began recruitment in May 2014 and was closed in May 2015. The follow-up period is currently underway.
